# Putative IL-10 Low Producer Genotypes Are Associated with a Favourable Etanercept Response in Patients with Rheumatoid Arthritis

**DOI:** 10.1371/journal.pone.0130907

**Published:** 2015-06-24

**Authors:** Heiko Schotte, Bernhard Schlüter, Hartmut Schmidt, Markus Gaubitz, Susanne Drynda, Jörn Kekow, Peter Willeke

**Affiliations:** 1 Niels-Stensen-Kliniken, Franziskus-Hospital Harderberg, Georgsmarienhütte, Germany; 2 Centrum für Laboratoriumsmedizin, Universitätsklinkum Münster, Münster, Germany; 3 Akademie für Manuelle Medizin, Rheumatologie, Münster, Germany; 4 Klinik für Rheumatologie, Universität Magdeburg, Vogelsang / Gommern, Germany; 5 Medizinische Klinik D, Universitätsklinikum Münster, Münster, Germany; Nippon Medical School Graduate School of Medicine, JAPAN

## Abstract

Outcome predictors of biologic therapeutic drugs like TNF inhibitors are of interest since side effects like serious infections or malignancy cannot be completely ruled out. Response rates are heterogeneous. The present study addressed the question whether in patients with rheumatoid arthritis (RA) interleukin-10 (IL-10) promoter genotypes with potential relevance for IL-10 production capacity are associated with response to long-term treatment with etanercept. Caucasian RA patients that, according to the EULAR criteria, responded well (n = 25), moderately (n = 17) or not (n = 8) to etanercept therapy (median 36 months, range 4–52), and 160 matched controls were genotyped for the IL-10 promoter SNPs -2849 G>A (rs6703630), -1082 G>A (rs1800896), -819 C>T (rs1800871) and -592 C>A (rs1800872). Haplotypes were reconstructed via mathematic model and tested for associations with disease susceptibility and therapy response. We identified the four predominant haplotypes AGCC, GATA, GGCC, and GACC in almost equal distribution. Patients that responded well carried the putative IL-10 low producer allele -2849 A or the haplotypes AGCC and GATA (RR 2.1 and 4.0, respectively; 95% CI 1.1–4.0 and 1.1–14.8), whereas an unfavourable response was associated with carriage of the putative high producer haplotype GGCC (RR 1.9, 95% CI 1.1–3.3). No significant associations of alleles or haplotypes with disease susceptibility were observed. In RA, a low IL-10 production which is genetically determined rather by haplotypes than by SNPs may favour the response to etanercept treatment. Iatrogenic blockade of TNF may reveal proinflammatory effects of its endogeneous antagonist IL-10. Further studies are needed to correlate these genetic findings to direct cytokine measurements.

## Introduction

The introduction of tumor necrosis factor (TNF) blocking agents into the therapy of rheumatoid arthritis (RA) is a story of remarkable success [1]. The efficacy of anti-TNF is comparable to or even better than methotrexate, and today, we are not aware of startling safety concerns. Additionally, antagonizing cytokines provided us with detailed insights into the pathophysiology of chronic inflammation [2]. However, about 30–40% of the patients fail to respond. Moreover, response to inhibitors with different mechanisms of action such as soluble receptors or monoclonal antibodies is heterogeneous. Compared to patients on conventional DMARD therapy, patients treated with anti-TNF seem to have a higher risk of serious infections, of tuberculosis, of infections by herpes zoster, and also the risk of melanoma seems to be slightly increased [3]. Thus, reliable predictors of therapy outcome are expedient, allowing for the rational selection of eligible patients.

Explicit immunological consequences of blocking TNF are not yet certain, as complex interactions within the cytokine network that support the ongoing inflammation are not fully understood. However, the balance of pro- and anti-inflammatory cytokines has been attributed an important role [4, 4]. In contrast to TNF-alpha, interleukin-10 (IL-10) is considered to mediate down-regulation of the inflammatory cascade, as it inhibits the activation and effector functions of T cells, macrophages, and monocytes [5, 6]. In particular, it acts as a negative autocrine regulator of TNF-alpha and other pro-inflammatory cytokines [7]. A direct anti-inflammatory potential of IL-10 in cartilage has been described [8]. On the other hand, effects of IL-10 are pleiotropic, as it stimulates B cell survival, proliferation, differentiation, and antibody isotype switching [6]. Elevated levels of IL-10 have been found in the serum and synovial fluid of RA patients, possibly contributing to the diminished T cell function and increased antibody and rheumatoid factor production [9]. In fact, IL-10 has been reported to activate B cells *in vitro* to promote autoantibody production like rheumatoid factor or antibodies against cyclic citrullinated peptide [10–12].

Interindividual variability in IL-10 secretion is determined to a large scale by genetic variance [13]. The IL-10 promoter contains polymorphic elements that combine to form 4 major haplotype families [14]. Linkage disquilibrium restricts three proximal single nucleotide polymorphisms (SNPs) at -1082 A>G, -819 T>C, and -592 C>A to assemble only three predominant haplotypes (ATA, ACC and GCC), which have been studied for their association with IL-10 production [15]. Homozygous carriers of GCC are considered as IL-10 high producers, emphasizing -1082 G as the most relevant allele [16–18]. Studies investigating a SNP at -2849 G>A have shown that G carriers significantly overproduce IL-10 [19, 20]. RA patients carrying -2849 G displayed higher autoantibody titres and a higher rate of joint destruction [21]. The relevance of this polymorphism as a part of extended haplotypes is not fully understood.

Based on theoretical considerations and experimental findings that attribute IL-10 a pathogenetic role in RA, the present study addressed the question whether genetic polymorphisms associated with the constitutional IL-10 production could additionally be related to anti-TNF therapy response in RA. Potential genetic predictors of anti-TNF response have been reported [22]. We previously observed an association of IL-10 promoter microsatellites, that have also been linked to susceptibility to RA, with the outcome under etanercept therapy [23, 24]. We now genotyped patients and healthy controls for the IL-10 promoter SNPs at -2849, -1082, -819, and -592, reconstructed haplotypes, and analyzed their association with response to long-term treatment with etanercept.

## Patients and Methods

### Study subjects

The study protocol was approved by the local independent ethics committee (Ethikkommission der Ärztekammer Westfalen-Lippe und der Medizinischen Fakultät der Westfälischen Wilhelms-Universität Münster). Patients and controls consented in written form to participate in the study. All 50 patients were German Caucasians and, as patient enrollement started before 2010, were obliged to fulfil the effective diagnostic criteria for rheumatoid arthritis as established by the American College of Rheumatology (ACR) in 1987 [25, 26]. Patients had to be adults with at least 18 years of age, 5 or more tender and 5 or more swollen joints out of 28, and an active disease reflected by a DAS28 of at least 3.3 [27]. Thirty-one (31) patients were recruited from the outpatient clinic for rheumatology, Department of Medicine, Münster University Hospital, Germany, and 19 patients from the Clinic of Rheumatology, University of Magdeburg, Vogelsang / Gommern, Germany. Thirty-eight (38) patients were females. Thirty-eight (38) patients were positive for rheumatoid factor. Baseline patient characteristics are depicted in **Table 1**. The patients had been pretreated either consecutively or simultaneously with in the median 3 disease modifying antirheumatic drugs (range 0–7). Fourty-one (41) patients had received methotrexate, 28 patients sulfasalazine, 28 patients gold salts, 25 patients antimalarials, 15 patients azathioprine, 12 patients leflunomide, 9 patients cyclosporine A, 3 patients cyclophosphamide, and, one patient each, infliximab, D-penicillamine or interferon-gamma. Ethnically, age- and sex-matched healthy controls (n = 160) derived from the Prospective Cardiovascular Münster (PROCAM) study [27]. Participants of this study were employees of Westphalian companies, with significant cardiovascular, pulmonary, metabolic, rheumatic, and renal disease being excluded before study admission by review of medical history and physical examination.

**Table 1:** Patient baseline characteristics.

	Median	Range
Age [years]	55	21–80
Disease duration [years]	6	1–39
Morning stiffness (minutes)	120	10–1440
ESR [mm/hour]	29	5–81
Tender joint count	12	6–27
Swollen joint count	15	5–25
DAS28	6.2	4.1–8.6
Steroid dosage [milligrams]	7.5	0–23

### Therapy

Recombinant human TNF receptor (p75)-Fc fusion protein (etanercept) was obtained from Pfizer Deutschland GmbH, Berlin, Germany. In Münster, 29 patients started therapy in randomized, placebo controlled trials for two (9 patients) or three months (20 patients), respectively. Within this period, they received 5 different doses of etanercept or placebo. The median etanercept dosage given in the controlled trials was 20 mg (range 10–100) per week. Thereafter, all 50 patients were treated with 25 mg etanercept as monotherapy subcutaneously twice a week for up to four years. Clinical evaluations comprised the calculation of a disease activity score (DAS28), a combined index based on a 28 tender and swollen joint count, erythrocyte sedimentation rate, and the patients global disease activity measured on a visual analog scale [28]. In Münster, follow-ups were performed before the start of therapy and then every three months. In Magdeburg, patients were examined at baseline, after 3 and 6 months and than every six months. Treatment response was assessed as defined by the EULAR criteria in an intention-to-treat analysis, with the last observation carried forward [29]. The median treatment period was 36 months, ranging from 4 to 52 months. We thereby surveyed a total of 148 patient-years under etanercept.

### Genotyping

DNA was extracted from EDTA-anticoagulated blood according to the standard protocol of Higuchi or by QIAamp DNA Blood Mini Kit (QIAGEN, Hilden, Germany) [30]. The following IL-10 promoter SNPs were genotyped: -2849 G>A (rs6703630), -1082 G>A (rs1800896), -819 C>T (rs1800871) and -592 C>A (rs1800872). Direct genotyping by real-time PCR (qPCR) was performed on an ABI 7900 HT (Applied Biosystems, Weiterstadt, Germany) in a 384 well format. Commercially available Custom TaqMan SNP Genotyping assays (c__1747363_10 for -592 C>A and c__1747362_10 for -819 C>T) and 2 x TaqMan Universal PCR Master Mix (Applied Biosystems, Weiterstadt, Germany) were used. Standard qPCR cycling conditions were applied: hot start hold for 10 minutes at 95°C followed by 40 PCR cycles consisting of two steps, denaturation at 92°C for 15 seconds, annealing and extension at 60°C for 1 minute. qPCR row data analyses and call of SNP types were done with the SDS 2.3 Software (Applied Biosystems, Weiterstadt, Germany). Because of repetitive Alu-sequences nearby the SNPs -2849 G>A and -1082 G>A no functional qPCR assays were available. We therefore used classical Sanger sequencing methods for these SNP determinations. In brief: AmpliTaq Gold PCR standard reactions (Applied Biosystems, Weiterstadt Germany, for primer sequences see **Table 2**) were performed in an Eppendorf Mastercycler (Eppendorf, Hamburg, Germany) using following PCR cycle conditions: hot start hold for 10 minutes at 95°C followed by 40 PCR cycles consisting of three steps, denaturation for 45 seconds at 95°C, annealing for 1 minute at 62°C and extension at 72°C for 60 seconds, followed by a final extension for 7 min. BigDye 3.1 standard DNA-sequencing reactions were performed in a Gene Amp PCR System 9700, followed by capillary electrophoresis done on an ABI 3730XL sequencer (Applied Biosystems, Weiterstadt, Germany). Raw data analyses and the call of SNPs were done with the SeqScape v2.7 software (Applied Biosystems, Weiterstadt Germany) [31].

**Table 2:** PCR and sequencing primers for IL-10 genotyping.

SNP primer name	Primer sequence
-1082 for	GTGCTATTCCCTGTTGGGACA
-1082 rev	ACACCATCTCCAGCACATAGA
-2849 for	CAAATCAATGGGGAAAGAATCA
-2849 rev	AATGTCAGGGAGAAGGGAGGT

Data show forward and reverse primer sequences used for genotyping of IL-10 promoter SNPs.

### Haplotype reconstruction and statistical analysis

Haplotypes were reconstructed using a Bayesian, coalescent theory-based method with the PHASE software (Version 2.1 for DOS) [32, 33]. The software incorporates a model that allows for recombination and decay of linkage disequilibrium with physical distance of alleles. Moreover, the type of polymorphism (SNP or multiallelic with/without stepwise mutation mechanism, respectively) is taken into account. Linkage disequilibria were calculated using the HaploView software (Version 4.2 for Windows) [34]. The software performs association tests in case control data to calculate D’ and r-squared (r = correlation coefficient) as metric parameters of linkage disequilibrium. Complete linkage required an r-squared of 1. Allele, geno- and haplotype frequencies in patients and controls as well as in patient subgroups with different therapy response were compared by Chi-squared test on a personal computer using the MedCalc software (Version 12 for Windows Vista). The strength of association was calculated as the odds ratio (OR) or relative risk (RR) and is presented with 95% confidence intervals (CI). For the multiple haplotypes a Monte Carlo simulation was applied using the CLUMP software (Version 2005 for DOS) [35]. The T4 statistics was used to test for significance which was assumed in case of a *P*-value < 0.05. For comparison of non-parametric data in paired samples we used the Wilcoxon signed-rank test and the MedCalc software.

## Results

### Clinical response to etanercept therapy

**Fig 1:**
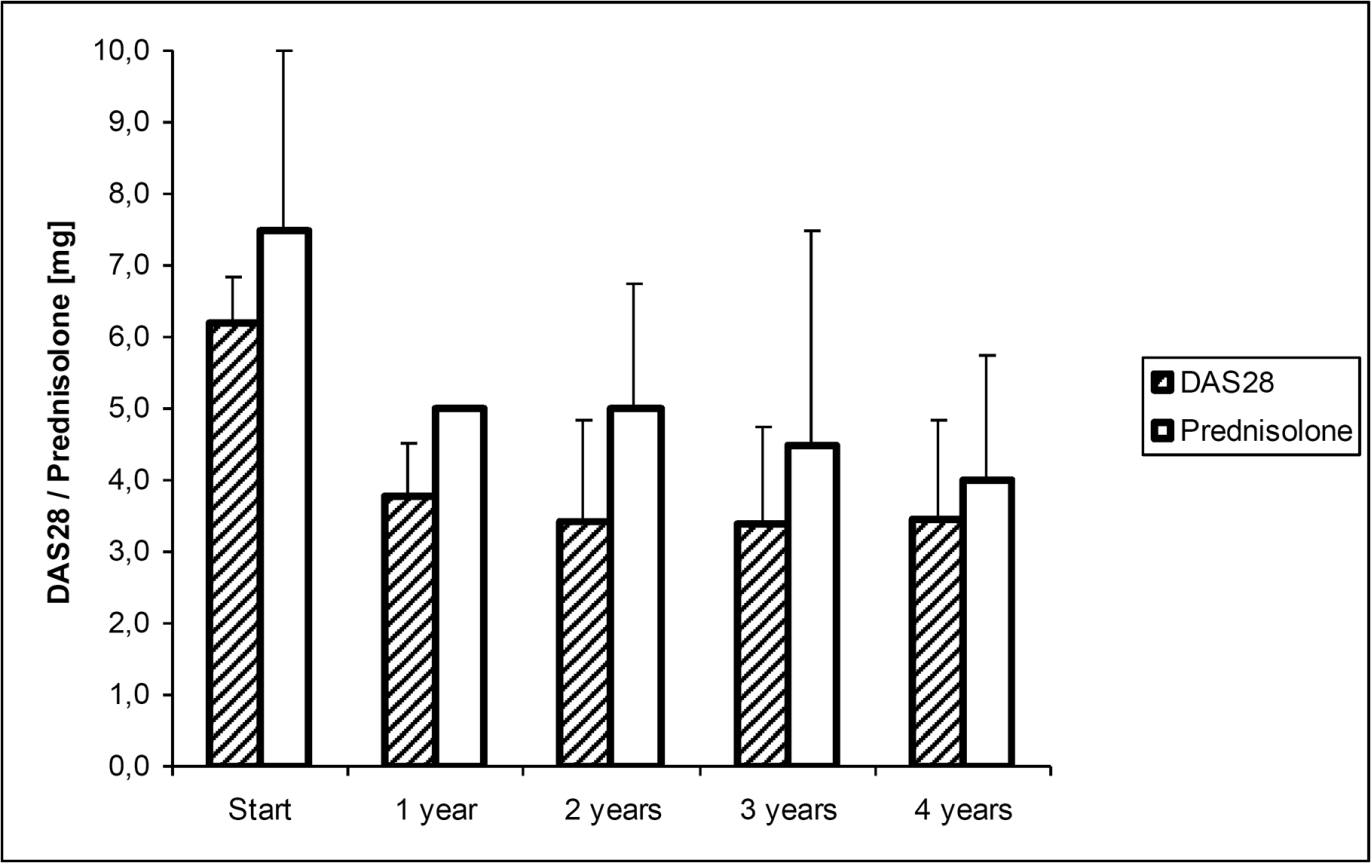
Course of DAS28 and prednisolone dosage under therapy. Bars represent the median (75% quartiles) of DAS28 and prednisolone dosage over time.

As shown in Fig 1, overall clinical response to etanercept was excellent. Within the first year, we registered a highly significant reduction in the median DAS28 from 6.2 to 3.8, and in the median steroid consumption from 7.5 to 5.0 mg prednisolone equivalent, both at *P* ≤ 0.0001. This reduction remained stable, as no further significant changes were observed thereafter. At start of therapy, 45 patients were at highly active disease (DAS28 > 5.1, maximum 8.6). At their final visit, 18 patients were in remission, 7 patients at low, 17 patients at moderate, and 8 patients at high disease activity. Thus, as defined by the EULAR criteria, 25 patients responded well, 17 patients moderately well, and 8 patients failed treatment. Apart from sporadic variation, this treatment response remained stable. Fourteen (14) patients stopped etanercept therapy within the study period. Reasons for discontinuation were ineffectiveness as assessed by the patients (n = 7), severe adverse events (n = 2), long distance to the study center (n = 2), scheduled surgery (n = 1), desire for pregnancy (n = 1), and non-compliance (n = 1). The severe adverse events that led to discontinuation of therapy were, in one patient each, a perforation of the sigmoid colon, and thrombocytopenia. Failure to etanercept therapy (= no response) was associated with a disease duration longer than 7 years (*P* = 0.0241). At their final visit, patients that responded well were significantly more often under prednisolone doses below 2.5 mg than patients who responded moderately or not (*P* = 0.0024). No significant associations were observed between response to therapy and patients sex, age at study entry, positivity for rheumatoid factor, number of previous DMARD therapies, DAS28 or steroid dosage at start of therapy.

### Genotype distribution in patients and healthy controls

**Fig 2:**
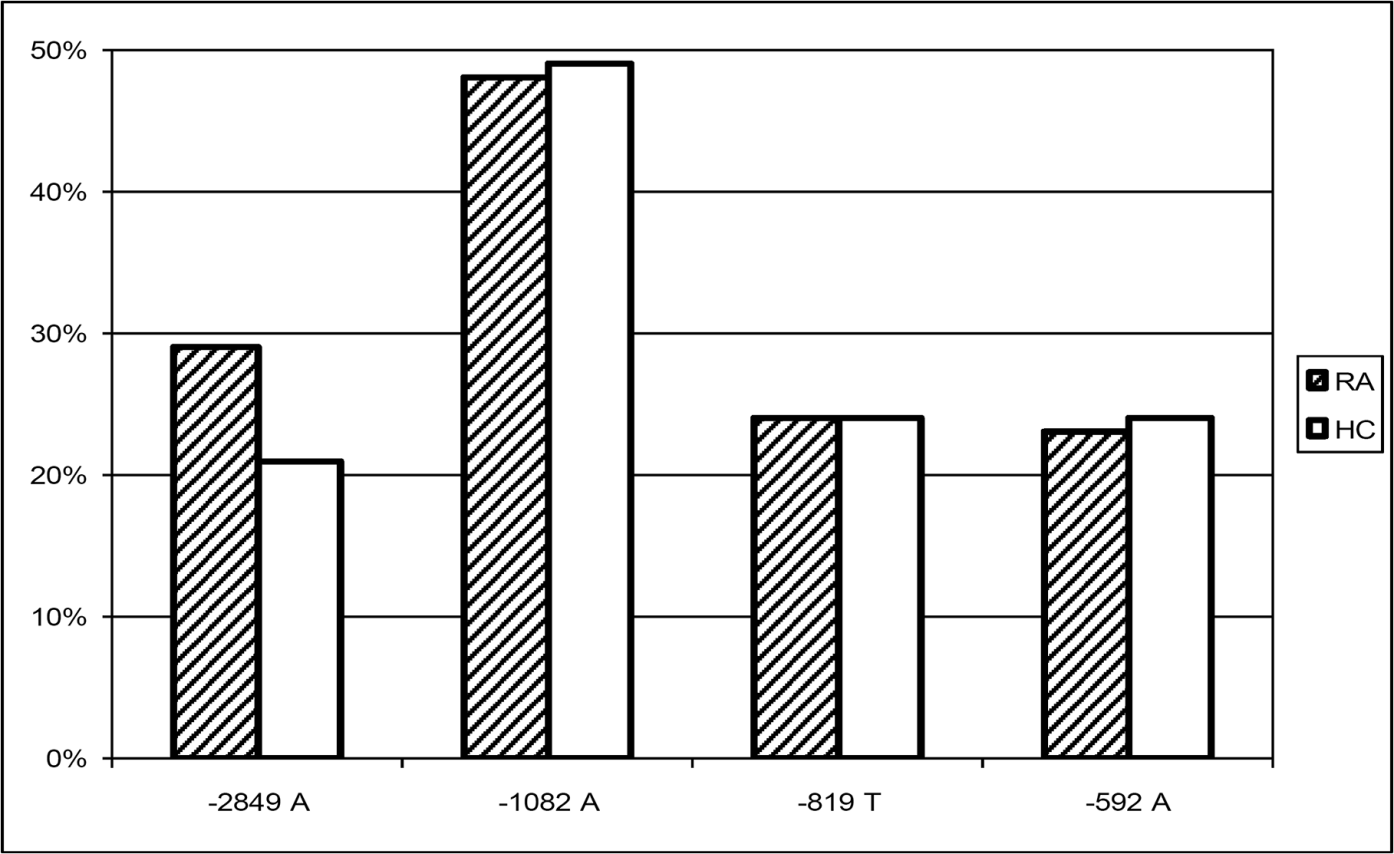
Allele distribution in RA patients and healthy controls. Bars represent minor allele frequencies of IL-10–2849 G>A, -1082 G>A, -819 C>T, and -592 C>A in RA patients (RA) and healthy controls (HC). No significant differences between RA patients and healthy controls were observed.

**Fig 3:**
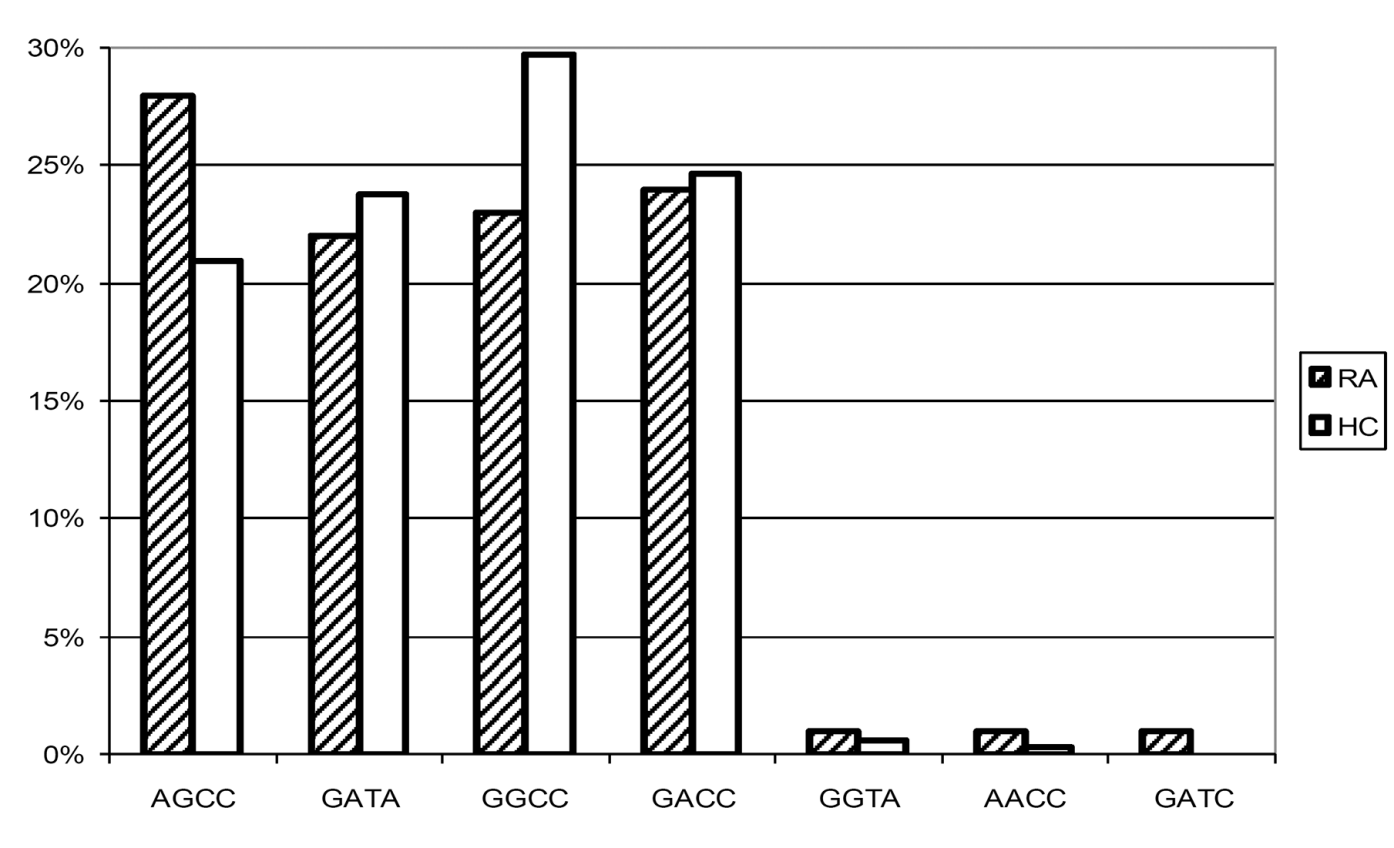
Haplotype distribution in RA patients and healthy controls. Bars represent haplotype frequencies of IL-10–2849 G>A, -1082 G>A, -819 C>T, and -592 C>A in RA patients (RA) and healthy controls (HC). No significant differences between RA patients and healthy controls were observed. Presumptive low / high IL-10 *in vitro* production capacity has been described for AGCC and GACC, respectively [14].

**Fig 4:**
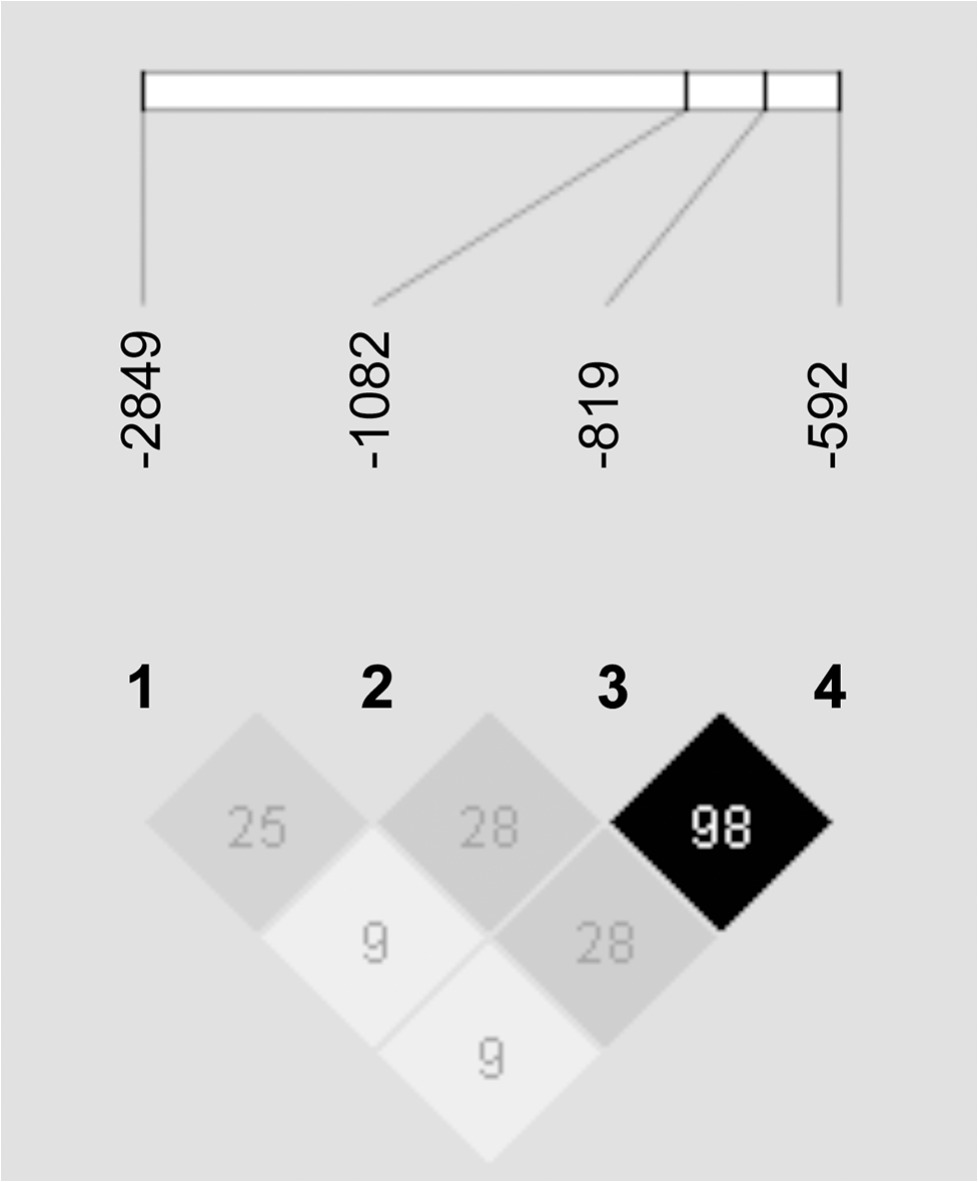
Linkage disequilibria in the IL-10 promoter region. Linkage disequilibria between IL-10–2849 G>A, -1082 G>A, -819 C>T, and -592 C>A, as reflected by r-squared values (Haploview 4.2) [38].

The minor allele frequencies of the four SNPs under study in RA patients and healthy controls are shown in Fig 2. Although we observed a trend of the -2849 A allele being more frequent in the RA patients, this association was not significant (χ^2^ = 2.16; *P* = 0.1514). This held true for the genotype distribution, as -2849 AA and AG genotypes were not overrepresented in the RA patients. Disease associations with the other alleles under study were neither found. Additionally, allele frequencies of -2849 were not associated with positivity for the rheumatoid factor (χ ^2^ = 0.08; *P* = 0.7840). Fig 3 depicts the haplotypes reconstructed using PHASE 2.1 [36, 37]. This method resulted in a median phase probability of 100%. Ninety-five (95) percent of the reconstructed phases had a probability of greater than 97%. Linkage disequilibria between the four loci under study were calculated with Haploview 4.2 and are shown in Fig 4. Linkage between -819 C>T and -592 C>A was nearly complete (D’ 1.0; r-squared 0.98), as only CC and TA were noted as proximal haplotypes. Linkage between the other loci was less strong. However, -2849 A only occurred in combination with the proximal GCC haplotype (D’ 0.9, r-squared 0.25), and -1082 A with the proximal TA haplotype (D’ 0.9, r-squared 0.28). As a consequence, in the RA population as well as in the controls we observed the four predominant haplotypes AGCC, GATA, GACC, and GGCC with frequencies between 0.2 and 0.3. There was no significant difference in the haplotype distribution between the patients and the healthy controls (χ ^2^ (T4) = 3.47; *P* = 0.3930), neither AGCC was associated with susceptibility to, nor GGCC with prevention from RA.

### Clinical response in relation to genotype distribution

**Fig 5:**
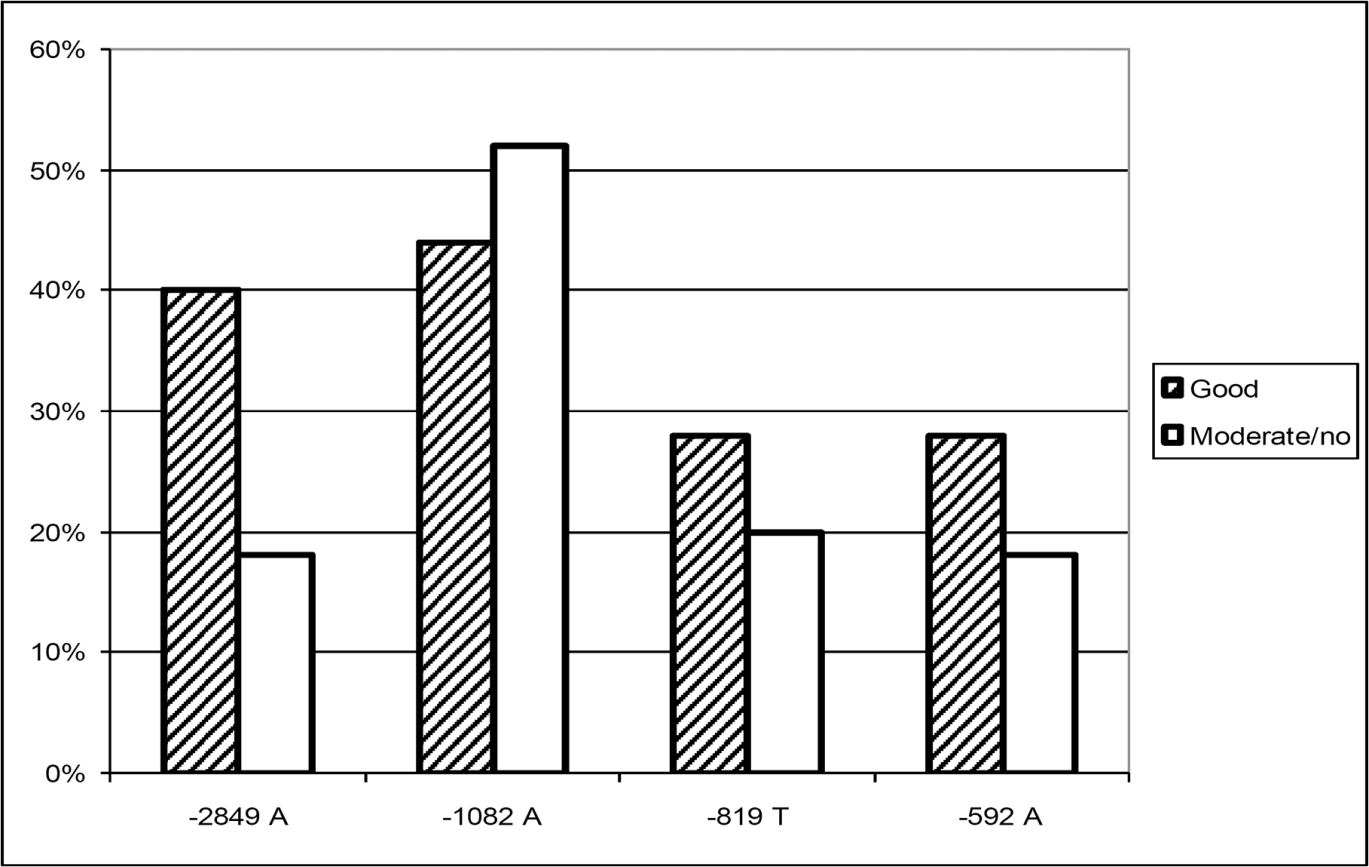
Allele distribution subject to etanercept response. Bars represent minor allele frequencies of IL-10–2849 G>A, -1082 G>A, -819 C>T, and -592 C>A in RA patients responding well, or moderately/not to etanercept therapy. -2849 A has been attributed a low IL-10 production capacity *in vitro* [19, 20].

**Fig 6:**
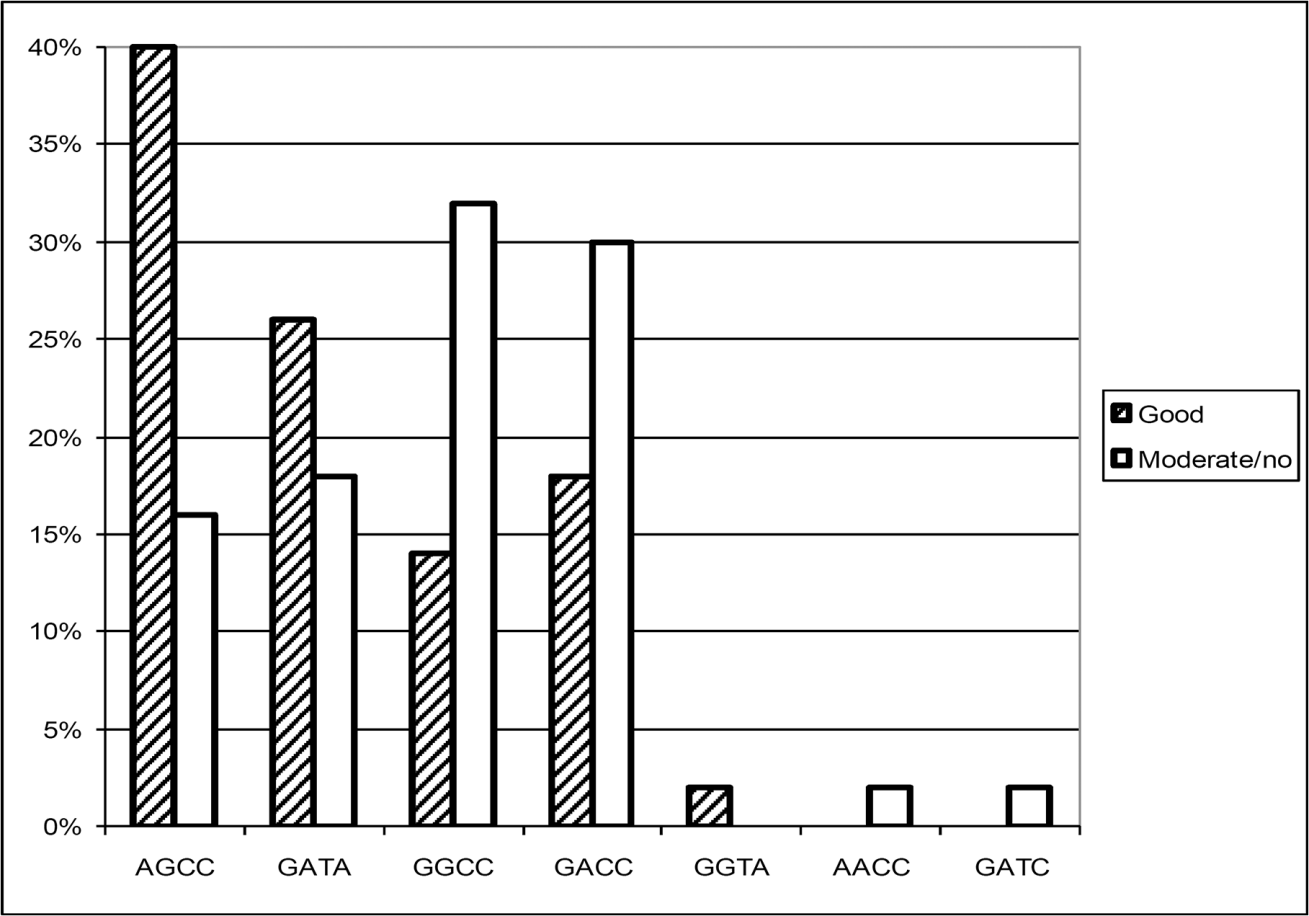
Haplotype distribution subject to etanercept response. Bars represent haplotype frequencies of IL-10–2849 G>A, -1082 G>A, -819 C>T, and -592 C>A in patients with different etanercept response. Presumptive low / high IL-10 *in vitro* production capacity has been described for AGCC and GACC, respectively [14].

The analysis of etanercept response relative to the four IL-10 promoter SNPs revealed a strong association of a favourable outcome under etanercept treatment with the putative IL-10 low producer -2849 A allele, which is shown in Fig 5 (OR 3.0, 95% CI 1.2–7.6). The relative risk of these well responding patients to carry the IL-10–2849 A allele is shown in **Table 3**. By contrast, the SNPs IL-10–1082, -819, and -592 were not independently associated with the therapy outcome. A closer inspection of the haplotypes as done in Fig 6 revealed an association of a good response with AGCC (OR 3.5; 95% CI 1.4–9.0) and of a moderate or no response with GGCC (OR 2.9; 95% CI 1.1–7.8). The maximum chance to respond favourably was observed when patients carried either AGCC *or* GATA, whereas patients responded moderately or not when carrying GGCC (**Table 3; S1 File**).

**Table 3:** Etanercept response and IL-10 promoter genotypes.

Allele/haplotype	N° in responders	N° in moderate / non-responders	Relative risk	to respond	95% CI
-2849 A	17	8	2.1	well	1.1–4.0
AGCC *or* GATA	23	14	4.0	well	1.1–14.8
GGCC	6	14	1.9	moderately / not	1.1–3.3

Data represent relative risk / 95% confidence interval of therapy response subject to carriage of distinct alleles or haplotypes. In the study population, 25 patients responded well, and 25 patients moderately or not.

## Discussion

The introduction of TNF blocking agents like the soluble TNF receptor-Fc fusion protein etanercept has been a milestone in the treatment of patients with chronic inflammation such as RA. Despite considerable clinical efficts, the risk of severe infection or distinct forms of malignancy cannot completely be ruled out [3]. Not all the patients respond equally well, and biologic DMARDs are of surpassing costs. Thus, reliable predictors of therapy outcome would be, without a doubt, beneficial, allowing for an adequate patient selection.

The RA population under study is representative with respect to clinical presentation and demographic data. Most of the patients were tested positive for the rheumatoid factor. Despite a considerable disease duration at start of therapy, the majority was at highly active disease, and most of them had been heavily pretreated. Response to therapy in more than 80% of the patients, which was paralleled by a highly significant reduction in the steroid consumption, is thus compelling. Surprisingly, the response went along with a shorter disease duration, but not with the disease activity at start of therapy. Of note, with one perforation of the sigmoid colon we observed a life threatening adverse event that again emphasises the need to monitor the patients for evidence of incipient complications like infection.

The principal finding of our study is the association of a favourable etanercept response with genotypes that have previously been linked to low IL-10 production capacity *in vitro* [20, 39]. This observation holds true for the -2849 G>A SNP and may be confirmed when analysing the extended haplotypes. Here we observed a haplotype–response relation, as a function of the presumptive *in vitro* IL-10 secretion capacity that has been described in one study [14]. Admittedly, the exact correlation of the IL-10 secretion capacity and the extended haplotypes has not yet been definitively established. However, studies consistently demonstrated low IL-10 production capacity for -2849 A containing haplotypes [20, 39]. By contrast, -2849 G containing haplotypes showed differential IL-10 production capacities, depending on the individual proximal haplotype structure. Based on comparable haplotype frequencies in our study population and in Dutch Caucasians, -2849 A presumably is linked to the microsatellite allele IL10.R3, and -2849 G to IL10.R2, respectively [14]. Eskdale et al. demonstrated an association of the lowest IL-10 secretion with the haplotype R3-GCC, whereas highest secretion was observed for R2-ACC. Nevertheless, we conclude that response to anti-TNF therapy may be favoured by a constitutionally low IL-10 production, although the functional assignment of the IL-10 promoter haplotypes has to be further characterized.

At a first glance, our finding seems to be suprising, as the majority of the IL-10 properties seem to be anti-inflammatory, and the balance between pro- and anti-inflammatory stimuli should actually determine the course of the disease [7]. On the other hand, IL-10 displays some pivotal pro-inflammatory characteristics that suit to support ongoing inflammation in the affected joints [6, 9, 40–42]. It has been established that IL-10 serum levels are elevated in patients with RA, and little efficacy has been seen in clinical studies performed with human recombinant IL-10 [43, 44]. Additionally, patients carrying the– 2849 G IL-10 high producer allele have previously been shown to suffer from more severe disease [21]. Thus, a low IL-10 production capacity may be associated with a milder course of the disease. The mere balance between TNF-alpha and IL-10 is not necissarily the crucial determinant in RA, especially when a therapeutic TNF blockade is applied, revealing pro-inflammatory effects of its endogeneous antagonist IL-10. Nevertheless, this hypothesis derives from genetic data, which actually have to be confirmed by measurement of cytokine levels.

With regard to susceptibility to RA the data from our small study population do not provide evidence that constitutionally different IL-10 production may be involved. This applies to the isolated SNPs as well as to the reconstructed haplotypes, and it matches previous reports [21]. Of interest, we observed a trend of -2849 A found slightly more often in our selected and, without a doubt, severely affected patient cohort. As an explanation may serve that a genetically determined low IL-10 production may result in an attenuated endogenous anti-inflammatory status, giving rise to an unreined pro-inflammatory activity of TNF-alpha.

As in previous studies, complexity of the IL-10 promoter with respect to functional aspects is reflected by the fact that the observed associations are generally stronger when the analysis is extended to haplotypes [31]. Thus, substantial information is gained when the structure of genetic elements is resolved to haplotypes rather than being restricted to the single nucleotide polymorphisms. The IL-10 promoter is suitable for this observation, as the number of observed haplotypes is, due to extensive linkage disequilibrium, restricted to half of the number expected. Additionally, inadequate implication of haplotype effects may account for negative results of large genome wide association studies investigating the association of SNPs with the anti-TNF response [45, 46].

In summary, our data provide evidence that putative IL-10 low-producer genotypes are associated with a favourable response to etanercept therapy in patients with RA. The restricted number of haplotypes within the IL-10 promoter qualifies the SNPs under study as accessible predictors of therapy outcome that could amend patient selection criteria. However, the associations described have to be further confirmed. Future studies should focus not only on therapy response, but especially on adverse events.

## Key Messages

IL-10 promoter SNP genotypes have previously been related to the cytokine production capacity *in vitro*. We observed an association of putative IL-10 low producer genotypes with a favourable etanercept response in patients with rheumatoid arthritis. This may prospectively help to identify patients that are eligible for an anti TNF therapy.

## Supporting Information

S1 File Data set Schotte. The data set contains genotyping results, clinical and demographic characteristics of patients and controls, and results of the statistical evaluation.
(XLS)

## References

[1] Aaltonen KJ, Virkki LM, Malmivaara A, Konttinen YT, Nordstrom DC, Blom M. (2012) Systematic review and meta-analysis of the efficacy and safety of existing TNF blocking agents in treatment of rheumatoid arthritis. PLoS One 7: e30275 10.1371/journal.pone.0030275 22272322 PMC3260264

[2] Rigby WF. (2007) Drug insight: different mechanisms of action of tumor necrosis factor antagonists-passive-aggressive behavior? Nat Clin Pract Rheumatol 3: 227–233. 17396108 10.1038/ncprheum0438

[3] Ramiro S, Gaujoux-Viala C, Nam JL, Smolen JS, Buch M, Gossec L, et al (2014) Safety of synthetic and biological DMARDs: a systematic literature review informing the 2013 update of the EULAR recommendations for management of rheumatoid arthritis. Ann Rheum Dis 73: 529–535. 10.1136/annrheumdis-2013-204575 24401994

[4] Striz I, Brabcova E, Kolesar L, Sekerkova A. (2014) Cytokine networking of innate immunity cells: a potential target of therapy. Clin Sci (Lond) 126: 593–612. 24450743 10.1042/CS20130497

[5] Wanidworanun C, Strober W. (1993) Predominant role of tumor necrosis factor-alpha in human monocyte IL-10 synthesis. J Immunol 151: 6853–6861. 8258695

[6] Moore KW, de Waal MR, Coffman RL, O'Garra A. (2001) Interleukin-10 and the interleukin-10 receptor. Annu Rev Immunol 19: 683–765. 11244051 10.1146/annurev.immunol.19.1.683

[7] Chernoff AE, Granowitz EV, Shapiro L, Vannier E, Lonnemann G, Angel JB, et al (1995) A randomized, controlled trial of IL-10 in humans. Inhibition of inflammatory cytokine production and immune responses. J Immunol 154: 5492–5499. 7730651

[8] Schulze-Tanzil G, Zreiqat H, Sabat R, Kohl B, Halder A, Muller RD, et al (2009) Interleukin-10 and articular cartilage: experimental therapeutical approaches in cartilage disorders. Curr Gene Ther 9: 306–315. 19534651 10.2174/156652309788921044

[9] Cush JJ, Splawski JB, Thomas R, McFarlin JE, Schulze-Koops H, Davis LS, et al (1995) Elevated interleukin-10 levels in patients with rheumatoid arthritis. Arthritis Rheum 38: 96–104. 7818579 10.1002/art.1780380115

[10] Perez L, Orte J, Brieva JA. (1995) Terminal differentiation of spontaneous rheumatoid factor-secreting B cells from rheumatoid arthritis patients depends on endogenous interleukin-10. Arthritis Rheum 38: 1771–1776. 8849349 10.1002/art.1780381210

[11] Reparon-Schuijt CC, van Esch WJ, van Kooten C, Levarht EW, Breedveld FC, Verweij CL. (1998) Functional analysis of rheumatoid factor-producing B cells from the synovial fluid of rheumatoid arthritis patients. Arthritis Rheum 41: 2211–2220. 9870878 10.1002/1529-0131(199812)41:12<2211::AID-ART17>3.0.CO;2-O

[12] Reparon-Schuijt CC, van Esch WJ, van Kooten C, Schellekens GA, de Jong BA, van Venrooij WJ, et al (2001) Secretion of anti-citrulline-containing peptide antibody by B lymphocytes in rheumatoid arthritis. Arthritis Rheum 44: 41–47. 11212174 10.1002/1529-0131(200101)44:1<41::AID-ANR6>3.0.CO;2-0

[13] Westendorp RG, Langermans JA, Huizinga TW, Elouali AH, Verweij CL, Boomsma DI, et al (1997) Genetic influence on cytokine production and fatal meningococcal disease. Lancet 349: 170–173. 9111542 10.1016/s0140-6736(96)06413-6

[14] Eskdale J, Keijsers V, Huizinga T, Gallagher G. (1999) Microsatellite alleles and single nucleotide polymorphisms (SNP) combine to form four major haplotype families at the human interleukin-10 (IL-10) locus. Genes Immun 1: 151–155. 11196662 10.1038/sj.gene.6363656

[15] Turner DM, Williams DM, Sankaran D, Lazarus M, Sinnott PJ, Hutchinson IV. (1997) An investigation of polymorphism in the interleukin-10 gene promoter. Eur J Immunogenet 24: 1–8. 10.1111/j.1365-2370.1997.tb00001.x 9043871

[16] Edwards-Smith CJ, Jonsson JR, Purdie DM, Bansal A, Shorthouse C, Powell EE. (1999) Interleukin-10 promoter polymorphism predicts initial response of chronic hepatitis C to interferon alfa. Hepatology 30: 526–530. 10421663 10.1002/hep.510300207

[17] Lazarus M, Hajeer AH, Turner D, Sinnott P, Worthington J, Ollier WE, et al (1997) Genetic variation in the interleukin 10 gene promoter and systemic lupus erythematosus. J Rheumatol 24: 2314–2317. 9415634

[18] Rood MJ, Keijsers V, van der Linden MW, Tong TQ, Borggreve SE, Verweij CL, et al (1999) Neuropsychiatric systemic lupus erythematosus is associated with imbalance in interleukin 10 promoter haplotypes. Ann Rheum Dis 58: 85–89. 10343522 10.1136/ard.58.2.85 PMC1752835

[19] Westendorp RG, van Dunne FM, Kirkwood TB, Helmerhorst FM, Huizinga TW. (2001) Optimizing human fertility and survival. Nat Med 7: 873 11479599 10.1038/90868

[20] de Jong BA, Westendorp RG, Eskdale J, Uitdehaag BM, Huizinga TW. (2002) Frequency of functional interleukin-10 promoter polymorphism is different between relapse-onset and primary progressive multiple sclerosis. Hum Immunol 63: 281–285. 12039409 10.1016/s0198-8859(02)00369-5

[21] Lard LR, van Gaalen FA, Schonkeren JJ, Pieterman EJ, Stoeken G, Vos K, et al (2003) Association of the -2849 interleukin-10 promoter polymorphism with autoantibody production and joint destruction in rheumatoid arthritis. Arthritis Rheum 48: 1841–1848. 12847677 10.1002/art.11160

[22] Prajapati R, Plant D, Barton A. (2011) Genetic and genomic predictors of anti-TNF response. Pharmacogenomics 12: 1571–1585. 10.2217/pgs.11.114 22044414

[23] Eskdale J, McNicholl J, Wordsworth P, Jonas B, Huizinga T, Field M, et al (1998) Interleukin-10 microsatellite polymorphisms and IL-10 locus alleles in rheumatoid arthritis susceptibility. Lancet 352: 1282–1283. 9788463 10.1016/S0140-6736(05)70489-X

[24] Schotte H, Schluter B, Drynda S, Willeke P, Tidow N, Assmann G, et al (2005) Interleukin 10 promoter microsatellite polymorphisms are associated with response to long term treatment with etanercept in patients with rheumatoid arthritis. Ann Rheum Dis 64: 575–581. 15345504 10.1136/ard.2004.027672 PMC1755447

[25] Arnett FC, Edworthy SM, Bloch DA, McShane DJ, Fries JF, Cooper NS, et al (1988) The American Rheumatism Association 1987 revised criteria for the classification of rheumatoid arthritis. Arthritis Rheum 31: 315–324. 3358796 10.1002/art.1780310302

[26] Aletaha D, Neogi T, Silman AJ, Funovits J, Felson DT, Bingham CO III, et al (2010) 2010 rheumatoid arthritis classification criteria: an American College of Rheumatology/European League Against Rheumatism collaborative initiative. Ann Rheum Dis 69: 1580–1588. 10.1136/ard.2010.138461 20699241

[27] Prevoo ML, van't Hof MA, Kuper HH, van Leeuwen MA, van de Putte LB, van Riel PL. (1995) Modified disease activity scores that include twenty-eight-joint counts. Development and validation in a prospective longitudinal study of patients with rheumatoid arthritis. Arthritis Rheum 38: 44–48. 7818570 10.1002/art.1780380107

[28] Assmann G, Schulte H. (1992) Relation of high-density lipoprotein cholesterol and triglycerides to incidence of atherosclerotic coronary artery disease (the PROCAM experience). Prospective Cardiovascular Munster study. Am J Cardiol 70: 733–737. 1519522 10.1016/0002-9149(92)90550-i

[29] van Gestel AM, Prevoo ML, van 't Hof MA, van Rijswijk MH, van de Putte LB, van Riel PL. (1996) Development and validation of the European League Against Rheumatism response criteria for rheumatoid arthritis. Comparison with the preliminary American College of Rheumatology and the World Health Organization/International League Against Rheumatism Criteria. Arthritis Rheum 39: 34–40. 8546736 10.1002/art.1780390105

[30] Higuchi R. (1989) Simple and rapid preparation of samples for PCR In: Ehrlich HA, editor. PCR-Technology, principles and applications for DNA amplification. New York: Stockton Press.

[31] Schotte H, Willeke P, Becker H, Poggemeyer J, Gaubitz M, Schmidt H, et al (2014) Association of extended interleukin-10 promoter haplotypes with disease susceptibility and manifestations in German patients with systemic lupus erythematosus. Lupus 23: 378–385. 10.1177/0961203314522334 24536045

[32] Stephens M, Smith NJ, Donnelly P. (2001) A new statistical method for haplotype reconstruction from population data. Am J Hum Genet 68: 978–989. 11254454 10.1086/319501 PMC1275651

[33] Stephens M, Donnelly P. (2003) A comparison of bayesian methods for haplotype reconstruction from population genotype data. Am J Hum Genet 73:1162–1169. 14574645 10.1086/379378 PMC1180495

[34] Barrett JC, Fry B, Maller J, Daly MJ. (2005) Haploview: analysis and visualization of LD and haplotype maps. Bioinformatics 21: 263–265. 15297300 10.1093/bioinformatics/bth457

[35] Sham PC, Curtis D. (1995) Monte Carlo tests for associations between disease and alleles at highly polymorphic loci. Ann Hum Genet 59: 97–105. 7762987 10.1111/j.1469-1809.1995.tb01608.x

[36] Stephens M, Smith NJ, Donnelly P. (2001) A new statistical method for haplotype reconstruction from population data. Am J Hum Genet 68: 978–989. 11254454 10.1086/319501 PMC1275651

[37] Stephens M, Donnelly P. (2003) A comparison of bayesian methods for haplotype reconstruction from population genotype data. Am J Hum Genet 73: 1162–1169. 14574645 10.1086/379378 PMC1180495

[38] Barrett JC, Fry B, Maller J, Daly MJ. (2005) Haploview: analysis and visualization of LD and haplotype maps. Bioinformatics 21: 263–265. 15297300 10.1093/bioinformatics/bth457

[39] Thye T, Browne EN, Chinbuah MA, Gyapong J, Osei I, Owusu-Dabo E, et al (2009) IL10 haplotype associated with tuberculin skin test response but not with pulmonary TB. PLoS One 4: e5420 10.1371/journal.pone.0005420 19412539 PMC2671601

[40] Perez L, Orte J, Brieva JA. (1995) Terminal differentiation of spontaneous rheumatoid factor-secreting B cells from rheumatoid arthritis patients depends on endogenous interleukin-10. Arthritis Rheum 38: 1771–1776. 8849349 10.1002/art.1780381210

[41] Reparon-Schuijt CC, van Esch WJ, van Kooten C, Levarht EW, Breedveld FC, Verweij CL. (1998) Functional analysis of rheumatoid factor-producing B cells from the synovial fluid of rheumatoid arthritis patients. Arthritis Rheum 41: 2211–2220. 9870878 10.1002/1529-0131(199812)41:12<2211::AID-ART17>3.0.CO;2-O

[42] Reparon-Schuijt CC, van Esch WJ, van Kooten C, Schellekens GA, de Jong BA, van Venrooij WJ, et al (2001) Secretion of anti-citrulline-containing peptide antibody by B lymphocytes in rheumatoid arthritis. Arthritis Rheum 44: 41–47. 11212174 10.1002/1529-0131(200101)44:1<41::AID-ANR6>3.0.CO;2-0

[43] Shrivastava AK, Singh HV, Raizada A, Singh SK, Pandey A, Singh N, et al (2015) Inflammatory markers in patients with rheumatoid arthritis. Allergol Immunopathol (Madr) 43: 81–87. 24656623 10.1016/j.aller.2013.11.003

[44] van Roon J, Wijngaarden S, Lafeber FP, Damen C, van de WJ, Bijlsma JW. (2003) Interleukin 10 treatment of patients with rheumatoid arthritis enhances Fc gamma receptor expression on monocytes and responsiveness to immune complex stimulation. J Rheumatol 30: 648–651. 12672180

[45] Umicevic MM, Cui J, Vermeulen SH, Stahl EA, Toonen EJ, Makkinje RR, et al (2013) Genome-wide association analysis of anti-TNF drug response in patients with rheumatoid arthritis. Ann Rheum Dis 72: 1375–1381. 10.1136/annrheumdis-2012-202405 23233654 PMC4169706

[46] Cui J, Stahl EA, Saevarsdottir S, Miceli C, Diogo D, Trynka G, et al (2013) Genome-wide association study and gene expression analysis identifies CD84 as a predictor of response to etanercept therapy in rheumatoid arthritis. PLoS Genet 9: e1003394 10.1371/journal.pgen.1003394 23555300 PMC3610685

